# Attenuation of cyclophosphamide toxicity by protective effects of curcumin in albino rats model

**DOI:** 10.1186/s12903-026-09975-z

**Published:** 2026-10-07

**Authors:** Noura Mohammed Bakr, Abeer Abdel Maksoud, Nehad Abdelmawgoud Ahmad

**Affiliations:** 1 Faculty of dental medicine for girls, Al-azhar university, Cairo, Egypt; 2 Faculty of dental medicine Fayoum university, Fayoum, Egypt; 3 Faculty of Dentistry, Mutah University, El Karak, Jordan

**Keywords:** Chemotherapy, Cyclophosphamide, Curcumin, TEM, Immunohistochemical study

## Abstract

**Background:**

Chemotherapy primarily treats cancer by using drugs to kill cancer cells. Curcumin, a natural adjuvant, shows promise in preventing side effects.

**Purpose:**

This study seeks to determine curcumin’s protective effect against cyclophosphamide toxicity.

**Materials and methods:**

Twenty-one male albino rats were used in this study, divided into three groups of seven. Group I received daily oral saline. Group II was given a single dose of CP (200 mg/kg) after seven days of saline with 0.5% CMC. Group III received CP (200 mg/kg) on day seven following seven days of CUR (30 mg/kg/day). At the experiment’s conclusion, the rats were euthanized under anesthesia, and their parotid salivary glands were collected.

**Results:**

The control group showed normal architecture. In group II, histological changes included necrosis of serous acini, striated duct, and atrophy of some acini cells. Group III displayed slight improvement in cell architecture. Cytochrome.

**Conclusion:**

The study examined Curcumin’s role in preventing Cyclophosphamide-induced changes.

## Introduction

One of the most popular treatment approaches for cancer is chemotherapy. Nevertheless, the medications currently used in chemotherapy only partially work and have serious adverse effects, such as myelosuppression, neurotoxicity, ototoxicity, gastrointestinal toxicity, cardiotoxicity, hepatotoxicity, etc. These serious side effects cause many patients to discontinue chemotherapy. Therefore, effective adjuvant strategies must be investigated in order to prevent and minimize the side effects caused by chemotherapy [1–3]. 

Perhaps the most widely used antineoplastic drug is cyclophosphamide, an alkylating agent with potent immunosuppressive and antineoplastic effects. More potent anti-cancer metabolites may be produced by the metabolism of anti-cancer medications, but these metabolites may also be a factor in the observed [4, 5].

The use of naturally occurring products in adjuvant therapy has shown encouraging promise in preventing side effects from chemotherapy. Turmeric’s active ingredient, curcumin (1,7-bis(4-hydroxy-3-methoxyphenyl)-1,6-heptadiene-3,5-dione), has shown strong antioxidant activity in vitro and in vivo [6]. Reactive oxygen and nitrogen species, such as hydroxyl and nitrogen dioxide radicals, can be effectively scavenged by curcumin. Additionally, curcumin has antiviral, hypolipidemic, anti-inflammatory, antioxidant, anti-diabetic, and anticarcinogenic qualities [7].

By scavenging reactive oxygen and nitrogen species, curcumin lowers oxidative stress-induced pathologies and inhibits inflammatory cytokines. Additionally, it prevents lipid peroxidation and encourages the production of different antioxidant and cytoprotective proteins [8]. It can also chelate oxidative metal ions, such as lead, protecting cells and preventing their buildup in tissues [9]. Because curcumin has such pleiotropic beneficial effects, the current study was conducted to see if the turmeric pigment could protect against cyclophosphamide-induced toxicity.

### Aim of the study

Assessment of curcumin’s ability to prevent cyclophosphamide toxicity.

## Materials and methods

### Chemicals and drugs

The medication cyclophosphamide The drug (1 g vial/injection form) was bought from a pharmacy. The brand is Endoxan (Baxter Oncology GmbH, Kantstrasse 2, D-33790 Halle, Germany) [10].

Curcumin powder was made from curcumin extract; BIO BASIC INC. sold high-purity curcumin powder. It was dissolved at a concentration of 30 mg/ml in distilled water. Oral doses of 30 mg/kg/day were given to the rats [11].

### Animals

Determine the sample size. A 95% confidence level, a 14.15% confidence interval, and a 5% P value were established using the statistical sample size calculator (www.Surveysystem.com). The software tells the user to leave it blank if the population size is large or unknown. Twenty-one was the computed total sample size. Consequently, each group in the study had seven rats.

Twenty-one male albino rats weighing 200–250 g and aged 5–6 months were used in this study. The animals were brought and kept in the Animal House at AL AZHAR University’s Faculty of Science. Each group was kept in a separate cage with unrestricted access to food, a temperature range of 22 to 24 °C, and a light-controlled environment with a 12:12 light-dark cycle. Before the experiment, rats were given a typical commercial pellet diet and allowed to acclimate for a week.

The experiment lasted for 21 days and the animals were split up into three groups of seven rats each:

1. Group I: The control group was given saline orally once a day via a blind-ended tube.
2. Group II (CP): On the seventh day, rats were given a single dose of CP (200 mg/kg, Sigma, St. Louis, MO, USA) after receiving saline containing 0.5% CMC orally for seven days [12].
3. Group III (CUR + CP): Rats were given a single dose of CP (200 mg/kg) on the seventh day after receiving CUR (300 mg/kg/day) [13] for seven days [12].
  1- The animals were put to death under anesthesia on the eighth day of the experiment, (animals were euthanized by an overdose of anesthetic thiopental sodium at dose of 50 mg/kg, i.p. (EIPICO, Ramadan City, Egypt), and samples of their parotid salivary glands were taken right away.

At the conclusion of the experiment, the parotid salivary gland was removed.

The tissues were embedded in paraffin wax and fixed in 10% formalin for histopathological analysis under light microscopy. Hematoxylin and eosin (H-E) staining was applied after the paraffin wax embedded specimens were cut into sections that were five µm thick and placed on slides. A Leica DFC280 light microscope and a Leica Q-Win Image Analysis system (Leica Micros Imaging Solutions Ltd, Cambridge, UK) were used to examine the tissue samples.

### Immunohistochemical study: cytochrome c

Cytochrome C is a small protein that plays a crucial role in the intrinsic pathway of apoptosis by being released from the mitochondrial membrane into the cytoplasm and initiating apoptotic pathways.

#### Steps of technique

Optiplus charged glass slides were used to cut paraffin sections.

After being deparaffinized in xylene, the sections were rehydrated for five minutes each using graded ethanol (100%, 95%, and 70%).

After being cleaned with distilled water, the slides were immersed in phosphate buffered saline (PBS) for five minutes.

A 3% hydrogen peroxide solution in methanol inhibited endogenous peroxidase activity for half an hour at room temperature.

Slides were submerged in 200 milliliters of 10 M citrate buffer (PH 6) after being thoroughly cleaned in PBS.

Sections were microwaved three times for five minutes each at 700 °C using a Smart Easy microwave.

After five minutes of distilled water washing, PBS was added to the slides.

A 1:40 dilution of one or two drops of mouse monoclonal antibody cytochrome c IGg was applied overnight at a humid room temperature.

After five minutes of distilled water washing, PBS was added to the slides.

Daminobenzidene (DAB) was applied for two to four minutes in order to develop the color.

Mayer’s hematoxylin was used as a counterstain after sections were placed in distilled water.

Sections were immersed in distilled water, counterstained with Mayer’s hematoxylin for one minute, and then cleaned with tap water. Twelve. The slides were immersed in two rounds of 95% alcohol and two rounds of absolute alcohol for three minutes each, followed by two three-minute intervals of absolute alcohol, mounted, and covered.

### Transmission electron microscope ultra thin section

Using a very sharp blade, tiny, one-millimeter-sized pieces of salivary gland were cut out and quickly fixed in a 3% phosphate buffered gluteraldehyde solution for one to two hours (primary fixation). The specimens were cleaned and fixed for one to two hours at 4˚C in 1% buffered osmium tetroxide. After dehydration in increasing ethanol concentrations (50%, 70%, and 90%), fresh epoxy resin was embedded in oven-dried gelatin capsules. To identify the target site, semithin Sects. (1–2 microns) were cut and stained with toluidine blue before being inspected under a light microscope. Glass knives and an ultramicrotome were used to cut ultrathin Sect. (0.06 microns), which were then placed on copper grids. At the National Research Center, Dokki, Cairo, Egypt, the transmission electron microscope (JEOL 1000) was used for the final examination at various magnifications, and the C.C.D electron microscope unit (TEM) was used to take pictures [14]. 

## Results

### Histological results

**Fig. 1:**
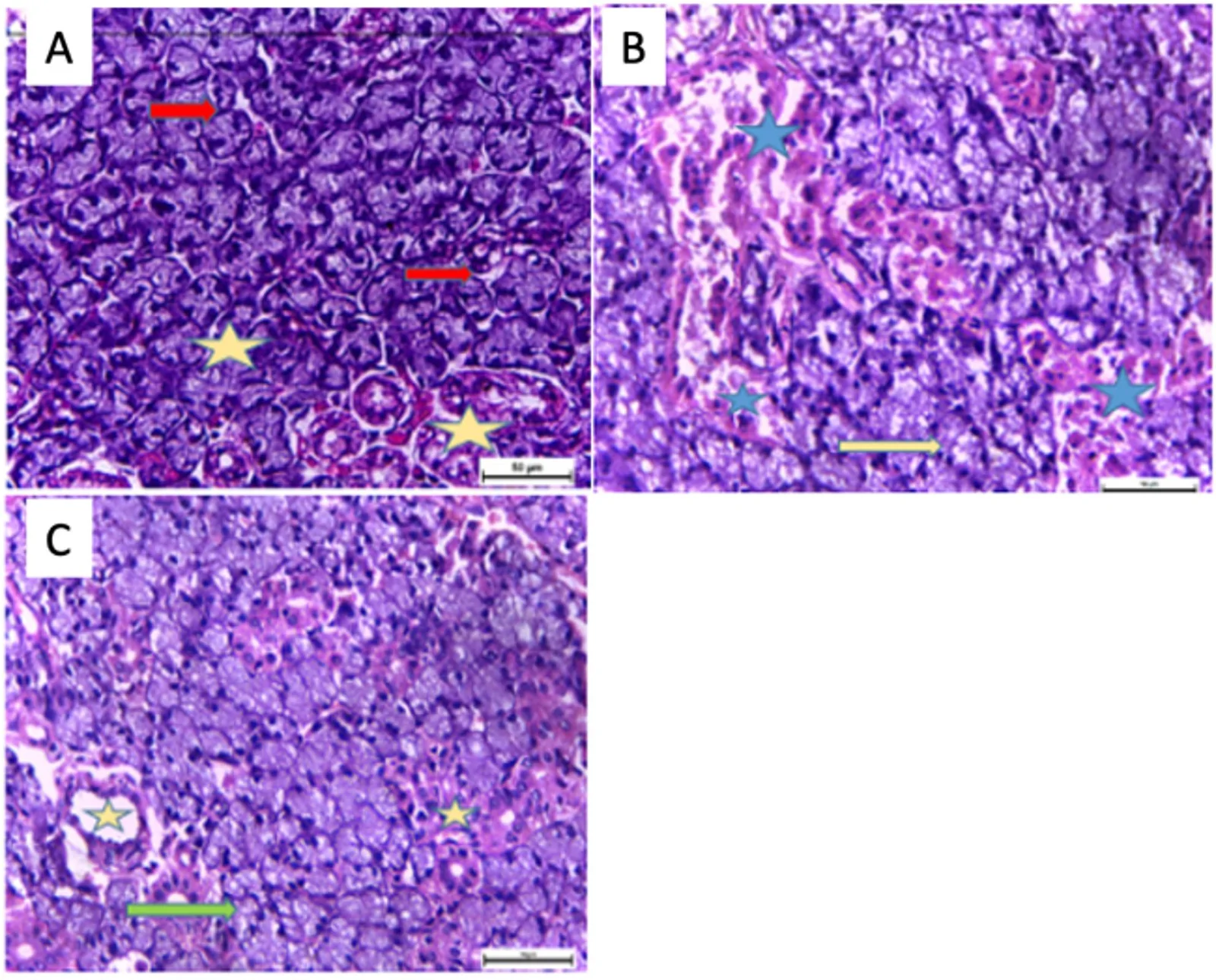
**A** Light microscopic examination, with H&E stain, of rats’ parotid salivary gland sections showed normal architecture represented by intact serous acini (red arrow), striated ducts (yellow stars) and interlobular ducts. **B** histological changes: include features of necrosis and atrophy of some serous acini, in addition to vacuolations in some acinus was seen (yellow arrow), In addition, there’s a loss of normal articheture of intralobular duct (striated duct) (green arrow) and degenerated interlobular duct (blue stars). **C** showed semi-normal appearance of serous acini (green arrow), mild degree of improvement of cells architecture of striated duct (yellow star) and Interlobular duct (yellow star) (**B**)

A light microscopic examination with H&E stain of rats’ parotid salivary gland sections from the control group revealed normal architecture, including intact serous acini, striated ducts, and interlobular ducts. Necrosis of serous acini, striated duct, atrophy of some acini cells, vacuolations in serous acini, and loss of normal articheture in the intralobular duct (striated duct) are among the histological alterations in group II. In comparison to group (II), group III showed a somewhat improved cell architecture and a semi-normal appearance of striated duct and serous acini (Fig. 1).

**Fig. 2:**
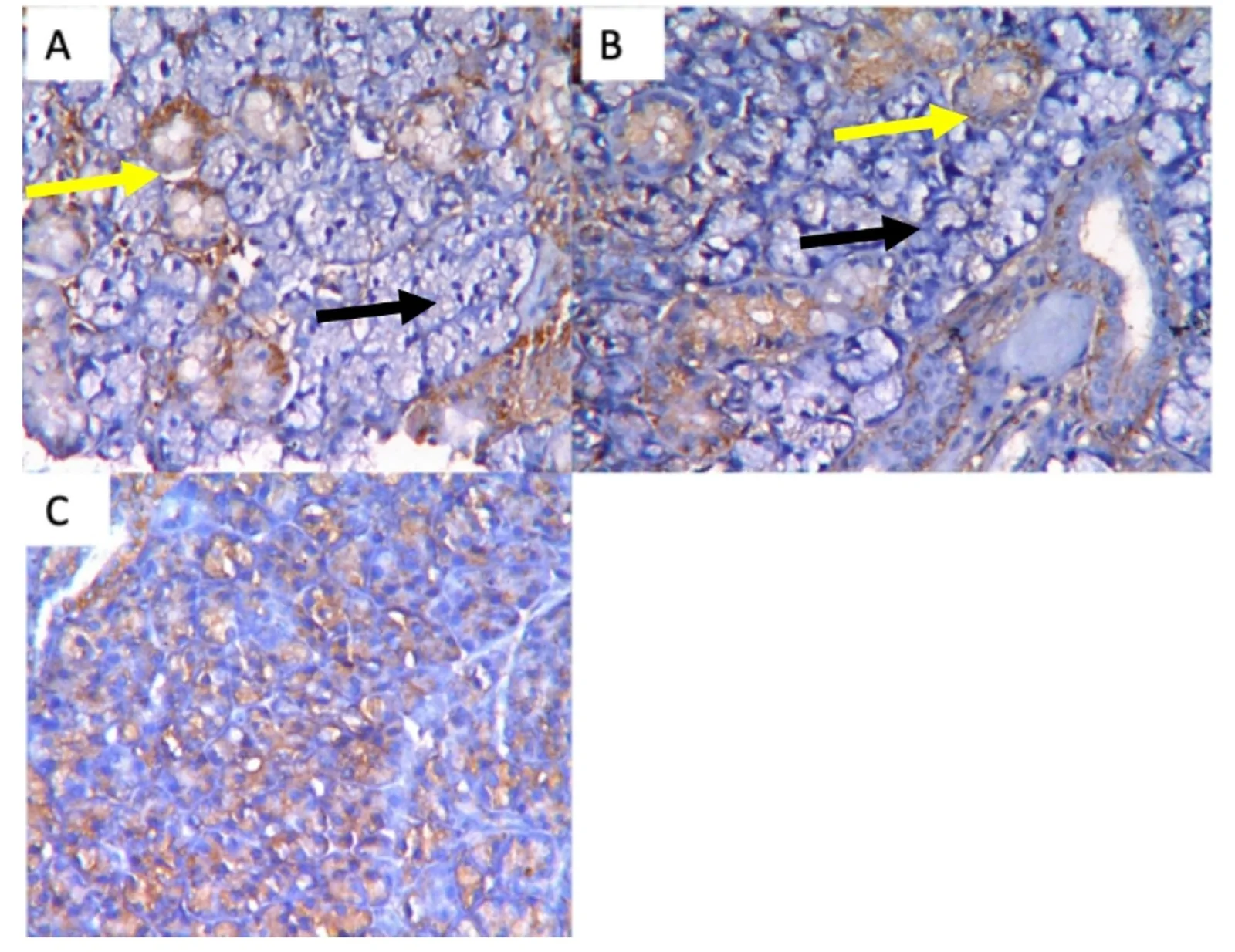
Photomicrographs of parotid salivary gland of all examined groups showing: (cytochrome C immunostaining × 200) (Group **A**): Control group showing no reaction in acini (black arrow) and very mild reaction in duct system (yellow arrow). Group **B**: The glands showed moderate to strong cytoplasmic immunoreactions in some serous acini and strong reaction in other acini. The striated duct showed stronger reaction to cytochrome c. Group **C**: The glands showed low +ve reaction to Cytochrome C in the lining epithelium of the ducts and negative reaction in acini

Cytochrome C immunostaining of Control group I revealed no reaction in acini and a mild reaction in the duct system. Group II glands exhibited moderate to strong cytoplasmic immunoreactions in some serous acini and a strong reaction in others. The striated duct reacted more strongly to cytochrome C, whereas the glands in group III reacted less positively to cytochrome C in the duct lining epithelium (Fig. 2).

**Fig. 3:**
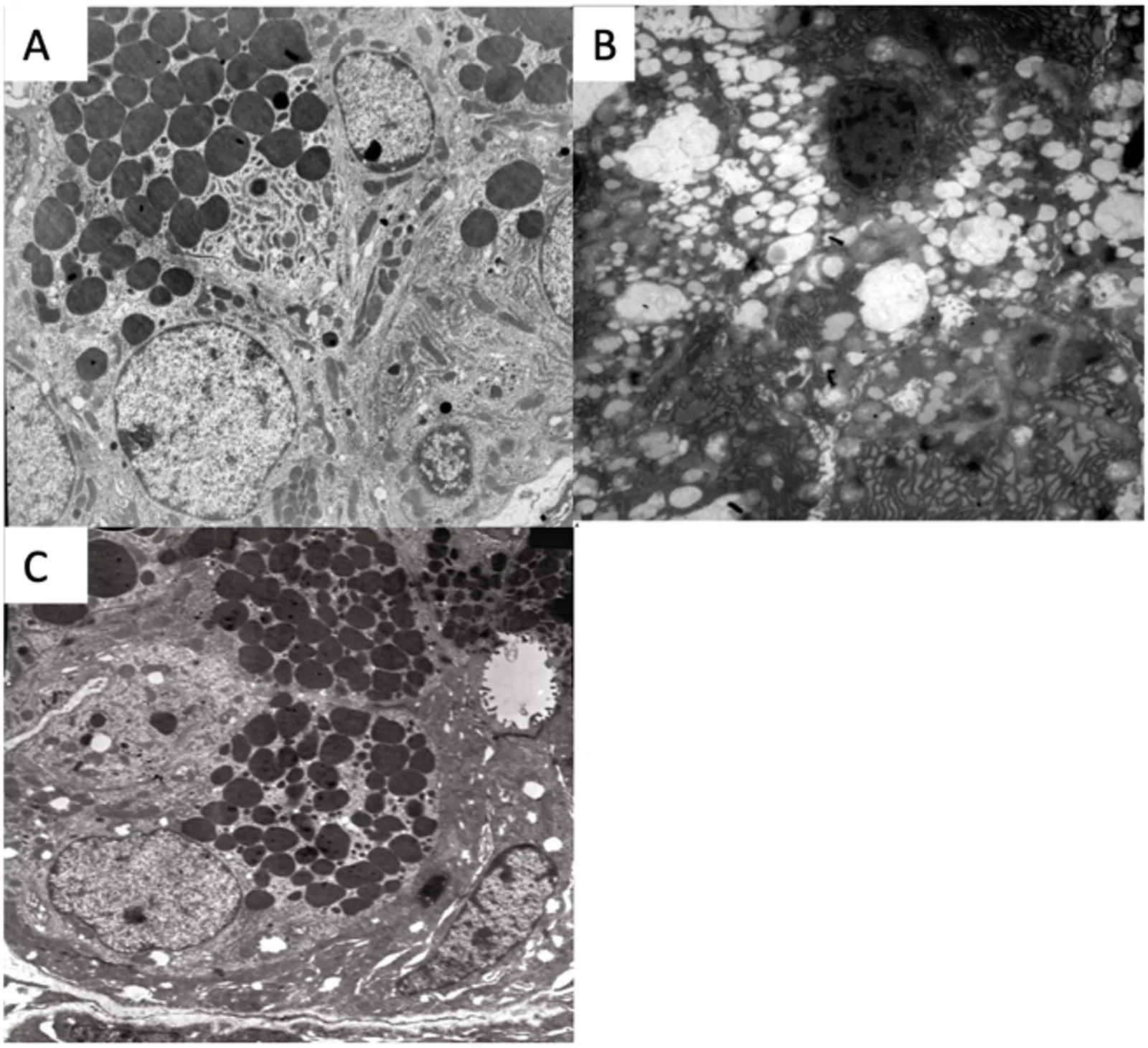
An electron micrograph of rat parotid salivary gland showing part of a serous cell. In (group **A**) showing nucleus and numerous secretory granules. In (group **B**) showing pyknotic nuclei with polymorphism, and numerous coalescing secretory granules. In (group **C**) showing decrease in signs of acinar degeneration (TEM- Orig. Mag. x 1000)

Ultrastructural analysis revealed that the serous secretory acini of the parotid salivary gland in group I (control group) had a spherical shape with a narrow lumen in the center. The serous acinar cells’ spherical, open-faced nuclei were located basally. (Fig. 3A).

Serous acinar cell degeneration was severe in group II. The size, shape, and location of the nuclei varied (polymorphism). chromatin condensation, shrinkage, and nuclear pyknosis. Many coalescing electron-lucent secretory granules were visible in the serous acinar cells’ cytoplasm. (Fig. 3B). There were fewer indications of acinar degeneration in group III when serous acinar cells were examined ultrastructurally. Numerous coalescing secretory granules were visible in the serous acinar cells’ cytoplasm, which resulted in a slight indentation of some nucleus on the periphery.(Fig. 3C). 

## Discussion

The ability of CUR to prevent the histological and ultrastructural changes caused by cyclophosphamide was investigated. Curcumin is a naturally occurring compound with strong anti-inflammatory and antioxidant qualities [15].

CP is a common immunosuppressive and antineoplastic that can be extremely toxic to several human and experimental animal organs.

The rat parotid gland underwent adverse histopathological and ultrastructural changes as a result of CP administration in the current investigation.

The degeneration of other cell organelles, like the Golgi apparatus, can also cause cytoplasmic vacuolations, which appear as empty spaces that are contained by TEM findings [16]. Cyclophosphamide’s oxidative stress effect actually causes several alterations, including lipid peroxidation, which harms biosystem cells [17]. Elsaied has demonstrated how oxidative action can lead to genetic alterations and DNA damage that trigger programmed cell death, or apoptosis [18].

Cyclophosphamide exerts its cytotoxic effects and stops cell division by damaging DNA. It is broken down internally by the liver’s cytochrome P450 system to produce acrolein, phosphoramide mustard, and other cytotoxic metabolites. Toxic acrolein accelerates apoptosis by inducing oxidative stress [19, 20]. 

Long-term CYP-induced ROS production can trigger stress signaling and pro-inflammatory pathways, which can then release pro-inflammatory cytokines like IL-1β [21]. 

Pro-inflammatory nuclear factor kappa B (NF-κB) inhibition is said to lower the expression of inducible nitric oxide synthase and inflammatory cytokines (interleukin-1β and − 6), which are the mechanisms behind colorectal cancer’s anti-inflammatory and antioxidative effects.

Additionally, it has been demonstrated that CRC raises detoxification enzyme gene expression and enzymatic activity [22]. 

Furthermore, CRC aids in the inhibition of ROS by increasing the activity of detoxification-related genes and enzymes such as glutathione S-transferase, quinone oxidoreductase 1, and NAD (P) H [23]. 

Some authors claim that curcumin’s ability to stop apoptosis and mitochondrial damage by obstructing the intrinsic apoptotic pathway is the source of its protective effect. Curcumin effectively reduces the dysregulation of the cellular redox balance, according to other authors [24, 25]. 

Rats in group I exposed to normal saline and group III showed mild or negative cytoplasmic pattern expression in the stromal cells between the acini, while rats in the group II showed strong cytoplasmic pattern expression, according to this study’s immunohistochemical analysis of cytochrome c expression in parotid salivary glands. According to a different study, selenium increases the expression of the BCL-2 protein, which has an anti-apoptotic effect [26, 27].

Curcumin reduces cyclophosphamide toxicity in albino rats by inhibiting oxidative stress and apoptosis. Curcumin treatment has been shown in studies to help restore antioxidant enzymes, reduce oxidative damage markers, and improve histopathological damage in various cyclophosphamide-affected organs [28]. 

Hence the ability of curcumin to stop cyclophosphamide-induced histological and ultrastructural alterations was examined.

## Conclusion

It is possible to draw the conclusion from this study that curcumin protects against cyclophosphamide toxicity.

## Data Availability

All data available with authors.

## Abbreviations

CP: Cyclophosphamide
CMC: Carboxymethylcellulose
CUR: Curcumin
TEM: Transmission electron microscope
